# Limited Associations Between 5-HT Receptor Gene Polymorphisms and Treatment Response in Antidepressant Treatment-Free Patients With Depression

**DOI:** 10.3389/fphar.2019.01462

**Published:** 2019-12-19

**Authors:** Taichi Ochi, Natalya M. Vyalova, Innokentiy S. Losenkov, Diana Z. Paderina, Ivan V. Pozhidaev, Anton J. M. Loonen, German G. Simutkin, Nikolay A. Bokhan, Svetlana A. Ivanova, Bob Wilffert

**Affiliations:** ^1^ Department of PharmacoTherapy, - Epidemiology & -Economics, Groningen Research Institute of Pharmacy, University of Groningen, Groningen, Netherlands; ^2^ Mental Health Research Institute, Tomsk National Research Medical Center, Russian Academy of Sciences, Tomsk, Russia; ^3^ Department of Cytology and Genetics, National Research Tomsk State University, Tomsk, Russia; ^4^ GGZ Westelijk Noord-Brabant, Policy Office for Quality and Innovation of Care (BZI), Halsteren, Netherlands; ^5^ Department of Psychotherapy and Psychological Counseling, National Research Tomsk State University, Tomsk, Russia; ^6^ Department of Psychiatry, Addictology and Psychotherapy, Siberian State Medical University, Tomsk, Russia; ^7^ School of Non-Destructive Testing and Security, Division for Control and Diagnostics, National Research Tomsk Polytechnic University, Tomsk, Russia; ^8^ Department of Clinical Pharmacy and Pharmacology, University of Groningen, University Medical Center Groningen, Groningen, Netherlands

**Keywords:** 5-HT receptor genes, treatment response, polymorphism, antidepressant, treatment-free depression

## Abstract

Major depressive disorder has become a prominent cause of disability, as lifetime prevalence has increased to ~15% in the Western world. Pharmacological effects of serotonin (5-hydroxytryptamine, 5-HT) are mediated through 5-hydroxytryptamine receptor (5-HTR) binding. Serotonin regulation of amygdala activity is attained through activation of three 5-HT2 family receptor subtypes, 5-HT2A, 5-HT2B, and 5-HT2C. Specifically, HT2A and the HT2C receptors have similar gross cerebral distribution and function, with higher constitutive activity found in HT2C than in HT2A. We investigated the possible association of 5-HTR gene polymorphisms to specific and non-specific antidepressant treatment responses in treatment-free patients in Siberia. 156 patients, aged between 18–70 years and clinically diagnosed with depressive disorders, were treated with antidepressants for 4 weeks. Patients were genotyped for a subset of 29 SNPs from the following 5-HT Receptor genes: HTR1A, HTR1B, HTR2A, HTR2C, HTR3A, HTR3B and HTR6. Primary outcome was measured by differences in Hamilton Depression Rating Scale (ΔHAM‐D 17) scores between baseline/week two, week two/week four and baseline/week four. Univariate linear regression was initially conducted to determine the 5-HTR SNPs to be studied within the multiple linear regression. Multiple linear regression analyses over the three time periods were conducted for ΔHAM‐D 17 with independent factors including: age, gender, depression diagnosis, antidepressant treatment and selected 5-HTR SNPs. We found improved ∆HAM-D 17 in patients taking tricyclic antidepressants (0–4 weeks: B = 4.85, p = 0.0002; 0–2 weeks: B = 3.58, p = 0.002) compared to patients taking SSRIs. Over the course of study, significant associations between 5-HT receptors SNPs and antidepressant response were not identified.

## Introduction

Major depressive disorder (MDD) is a complex, polygenic disease and a leading cause of disability affecting individuals across the globe (Kessler and Bromet, 2013). Although there are multiple antidepressant treatments, nearly a third of patients do not respond positively to the initial prescription (Bauer et al., 2013; Bauer et al., 2015). Efforts to personalize treatment by identifying genetic biomarkers associated with antidepressant response have been inconclusive. This may be due to the heterogeneous character of mood disorders but also to the large number of antidepressant drugs being investigated within the studies, leading to varied mechanisms of action and differing targets.

Currently, patients are prescribed selective serotonin reuptake inhibitors (SSRIs) as first line treatment of depression, targeting serotonin (5-HT) reuptake and increasing the level of 5-HT (Loonen and Ivanova, 2016). 5-HT receptors play a role in the mechanism of action of antidepressants, with the different receptor subfamilies affecting serotonergic activity (Fink and Göthert, 2007; Barnes and Neumaier, 2011). 5-HT receptors are categorized into seven families, 5-HT1 to 5-HT7, each mediating different physiological effects of 5-HT. 5-HT receptors are part of the G-protein coupled receptor (GPCR) superfamily, with the exception of 5-HT3 receptor, a ligand-gated ion channel (Barnes and Neumaier, 2011). Activation of 5-HT2 receptors regulates activity of projection neurons within the amygdala, where high activity is known to be associated with MDD. Within 5-HT2 receptors three receptor subtypes are subcategorized (5-HT2A, 5-HT2B, and 5-HT2C), playing an important role in the regulation of amygdala activity and amygdala-mediated effects, both behavioral and psychological (Bombardi, 2014). 5-HT2A and 5-HT2C receptors modulate dopamine output within the central nervous system and are targets for various mood disorder medications (Van Oekelen et al., 2003; Bubar and Cunningham, 2006). Curiously, 5-HT2C is the only receptor known to undergo RNA editing, achieving greater protein diversity than the other 5-HT receptors (Burns et al., 1997). 5-HT2B receptors, on the other hand, were found to have a transient action in embryogenesis, which suppression was found to lead to morphological defects, and peripheral tissues but its role in the adult brain was undetermined (Nebigil et al., 2001; Leysen, 2004).

In this study, we selected 5-HT receptor SNPs which were previously associated with mood disorders and known to affect 5-HT mechanism of action. Studies have linked 5-HT2C (759-C/T polymorphism) with weight gain (Pooley et al., 2004; Del Castillo et al., 2013; Higgins et al., 2013; Reynolds et al., 2014). Polymorphisms within the *HTR2C* gene have been previously associated with criminal behavior, antipsychotic drug-induced hyperprolactinemia and tardive dyskinesia (Toshchakova et al., 2018; Ivanova et al., 2017; Loonen et al., 2019; Pozhidaev et al., submitted). Further investigation on 5-HT receptor polymorphisms may build on previous findings to develop a better understanding of mental health disease and treatment side-effects.

Our current research builds on our previous investigations on the Russian depression cohort, expanding the genotype targets to encompass 5-HT receptor single nucleotide polymorphisms (SNPs). The prior studies investigated the possible relationship between *PRL* and *BDNF* genotypes and serum protein levels on our depression cohort. *BDNF* rs6265 and severity of depression were found to be associated in the initial observations (Losenkov et al., submitted). To follow-up the findings, patients were treated with antidepressants for 4 weeks to investigate the potential pharmacogenetic *PRL* and *BDNF* markers in antidepressant treatment response (Ochi et al., 2019).

Many patients within our cohort were prescribed SSRIs and SNRIs throughout the study, therefore in this investigation, additional genotyping was conducted to investigate 5-HT receptor SNPs’ roles in treatment response in the cohort. As with the previous study, we investigated the ‘true’ antidepressant response to determine whether associations between 5-HT receptor polymorphisms and antidepressant response are found. This stems from our hypothesis where antidepressant response is delayed roughly 2 weeks and specific response to treatment given should be noticeable in after the 2 weeks. We aim to identify the effect of 5-HT receptor SNPs on antidepressant efficacy within the total and the latter 2 weeks of the study.

## Methods

### Study Design and Patient Characteristics

Following the investigation of *PRL* and *BDNF* to antidepressant treatment, further genotyping was conducted on 5-HT receptor SNPs. 156 participants from the initial 186, aged 18-70 years old, were genotyped and included based on the clinical diagnosis of a depressive episode (single or recurrent, ICD: F32 or F33). Depressed patients were assessed with the 17-item Hamilton Depression Rating Scale (HAM-D 17) (Hamilton, 1960). The increase in the number of patients compared to the previous study (151 patients genotyped) stems from increased funds available to expand the scope of our study.

The study was carried out in accordance with the Code of Ethics of the World Medical Association (Declaration of Helsinki 1975, revised in Fortaleza, Brazil, 2013), and approved by the Institutional Medical Review Board (protocol 49 from 23.04.12). Participants were recruited from psychiatric departments of the Mental Health Research Institute, Tomsk National Research Medical Center and provided written informed consent. Patients were excluded from the study considering the following conditions: non-Caucasian ethnicity, schizophrenia, decompensated personality disorders, pregnancy, or any relevant gynecological or endocrine (thyroid) disorder, relevant pharmacological withdrawal symptoms, organic brain disorders (e.g., epilepsy, Parkinson’s disease), or treatment with antidopaminergic drugs (antipsychotic or antiemetic drugs).

After diagnosing, assessing depressive status and obtaining informed consent, antidepressant treatments were initiated to all patients within our cohort. Out of the studied 156 patients, 133 patients were treated with serotonin reuptake inhibitors [selective serotonin reuptake inhibitors (SSRIs) – 91, tricyclic antidepressants (TCAs) – 23, serotonin and noradrenaline reuptake inhibitors (SNRIs) – 14], 23 patients were treated with serotonin type 2 receptor antagonists (**Table 1**). Depressed patients were assessed with the 17-item Hamilton Depression Rating Scale (HAM-D 17) (Hamilton, 1960).

**Table 1:** Antidepressant medication taken by patients.

Class	Antidepressant	Patients	Median Dosage (mg)	IQR (mg)
SSRIs	**Total**	94		
	Sertraline	25	100	100
	Paroxetine	22	20	20
	Escitalopram	17	10	10–18.75
	Fluoxetine	12	20	20–40
	Fluvoxamine	12	100	100–150
	Citalopram	4	20	20
	Trazodone	1	300	
	Trazodone + Paroxetine	1	170	
TCAs	**Total**	25		
	Clomipramine	18	100	75–150
	Pipofezine	6	100	68.75–150
	Amitriptyline	1	150	
SNRIs	**Total**	14		
	Venlafaxine	10	150	150–225
	Duloxetine	4	60	60–105
Agomelatine	Agomelatine	12	50	25–50
NaSSa	**Total**	11		
	Mirtazapine	7	15	10–30s
	Mianserin	4	38	30–60

Antidepressant taken over the course of the 4 weeks by patients. IQR, interquartile range (25^th^–75^th^ percentile); SSRIs, selective serotonin receptor inhibitors; TCAs, tricyclic antidepressants; SNRIs, serotonin–norepinephrine reuptake inhibitors; NaSSAs, noradrenergic and specific serotonergic antidepressants.

A control cohort consisting of 143 healthy participants of both genders, aged from 18 to 60 years old, were enrolled as a reference for the association study. Written informed consent was obtained from the participants and was genotyped to determine the association of 5-HT receptor polymorphisms to depression. The control participants were excluded if they presented the following conditions: chronic physical pathology in exacerbation, pregnancy, or any relevant gynecological or endocrine (thyroid) disorder; relevant pharmacological withdrawal symptoms; or organic brain disorders (e.g., epilepsy, Parkinson’s disease) or mental disorders.

### Genotyping

DNA was isolated from the leukocytes in whole peripheral blood from patients using the standard phenol-chloroform micro method and mandatory condition of pre-freezing the blood. The DNA was genotyped for a subset of 29 SNPs from the following 5-HT receptor genes: *HTR1A*, *HTR1B*, *HTR2A*, *HTR2C*, *HTR3A*, *HTR3B*, and *HTR6* in the Laboratory of Genetics of the University of Groningen with the MassARRAY^®^ System (Agena Bioscience™) and in the Laboratory of Molecular Genetics and Biochemistry of the Mental Health Research Institute with “StepOnePlus” (Applied Biosystems) (**Supplementary Table 1**). Blood sampling method can be found in the previous study (Ochi et al., 2019).

### Statistical Analysis

To determine the effect of 5-HT Receptor gene SNPs to antidepressant response, the outcome was measured by the difference in HAM-D 17 score between entry and 2 weeks of treatment (ΔHAM-D 17 0–2 weeks), after two and 4 weeks of treatment (ΔHAM-D 17 2–4 weeks) and entry and 4 weeks of treatment (ΔHAM-D 17 0–4 weeks), with a greater ΔHAM-D 17 score denoting better clinical outcome. Normal distribution was tested utilizing the P-P plot. Due to the small number of homozygous patients for some SNP, patients were combined with heterozygous patients in the analysis to determine the effect of the allele and compared against the major allele.

Patient characteristics (i.e. age, sex, ΔHAM-D 17) were determined using descriptive statistics (**Table 2**). Due to the larger number of variables to our cohort size, univariate linear regression was conducted initially to determine the variables to be included in the multiple linear regression. As the patient cohort, consisted of 156 patients, we proceeded with 16 variables in the multiple linear regression, utilizing the one in ten rule (Harrell et al., 1984). Multiple linear regression was conducted to identify the independent factors associated with ΔHAM-D 17 between the three time periods, including age, sex, depression diagnosis, type of antidepressant taken and 5-HT receptor gene SNPs. To study specific antidepressants and SNP genotypes, dummy variables were created to determine the effect within each variable. Without the creation of the dummy variables, differentiation between the antidepressant medication and alleles would not have been investigated. Odds ratio (OR) was calculated for *HTR2C* SNPs, due to the hemizygous nature of the gene in males (as the gene is X-linked). Statistical analysis was conducted with SPSS software (release 25.0). The significance level for descriptive statistics and univariate statistical tests were p < 0.05 and for the multiple linear regression was p < 0.0031, after Bonferroni correction. Power analysis was conducted post-hoc utilizing G*Power (Faul et al., 2007; Faul et al., 2009).

**Table 2:** Patient characteristics.

Characteristics	Depressed Cohort (n = 156)	Control Cohort (n = 143)
**Total Number of Patients (%)**		
Male	20 (12.8%)	54 (37.7%)
Female	136 (87.2%)	89 (62.3%)
**Age in Years (Mean ± S.D.)**	49.4 ± 10.9	35.5 ± 12.4
Male	49.3 ± 9.3	34.1 ± 12.2
Female	49.5 ± 11.1	36.3 ± 12.5
**HAM-D 17 score (Mean ± S.D.)**		
At entry	24.3 ± 5.2	N/A
At 2 weeks	12.9 ± 4.9	
At 4 weeks	5.0 ± 3.9	
**Type of Depressive Episode (%)**		
Single	87 (55.8%)	N/A
Recurrent	69 (44.2%)	

Patient characteristics of Depression Cohort who were genotyped for 5-HT Receptor SNPs and Control Cohort. N/A for Control Cohort as participants did not have depression nor were monitored for HAM-D 17. Data is presented as percentage (%) of cohort and mean ± standard deviation (S.D.). HAM-D, Hamilton Depression Rating Scale 17 Score.

## Results

From the initial cohort, 156 patients consisting of 20 men and 136 women, aged 49.4 ± 10.9 years (mean ± standard deviation), were genotyped for the 5-HT receptor SNPs. Over the course of 4 weeks, HAM-D 17 was taken at entry (HAM-D 17 score: 24.3 ± 5.2), at 2 weeks (HAM-D 17 score: 12.9 ± 4.9) and at 4 weeks (HAM-D 17 score: 5.0 ± 3.9) (**Table 1**). The control cohort included 143 patients, comprising of 54 male and 89 women and aged 35.5 ± 12.4 years. The control cohort was genotyped for 5-HT2C receptor SNPs to determine the Odds Ratio for depression, when compared to the depression cohort.

*HTR1A* rs1800042 was not included in the analysis as it violated the Hardy-Weinburg Equilibrium distribution. Of the remaining 28 SNPs investigated, 13 of the SNPs had the minor allele combined with the heterozygous allele for analyses (**Supplementary Table 1**). As we were interested in determining which SNPs influenced antidepressant treatment response, the SNPs genotypes were not categorized into dummy variables for the univariate analyses.

### Linear Regression to Determine Covariate Associations With ∆HAM-D 17

Univariate linear regression was conducted for the 5-HTR SNPs to determine which SNPs were to be included in the multiple linear regression analyses of the three time periods for ∆HAM-D 17 (**Supplementary Table 2**). SNPs found to be significant in the univariate analyses, had a p-value lower than 0.3 or were found to be associated in the multiple linear regression where all 5-HTR SNPs were included into the multiple linear regression analysis.

For the multiple linear regression, the following covariates were included: gender, age, type of diagnosis, antidepressant treatment, *HTR1A* rs6295, rs749099, *HTR1B* rs6296, *HTR2A* rs7997012, rs1928040, rs6312, *HTR2C* rs5946189, rs6318, rs17326429, *HTR3A* rs33940208, *HTR3B* rs1176744, and *HTR6* rs1805054. To determine the effect of a SNP’s genotype on antidepressant response and type of antidepressant treatment taken, dummy variables were created for the SNPs and antidepressants treatments. Dummy variables provide an opportunity to determine the effect of the genotypes within SNPs and see which allele affected antidepressant response. Similarly, dummy variable for antidepressant treatments determine the effect treatments have on ∆HAM-D 17, when referenced to SSRIs. Bonferroni correction was imputed by taking the standard alpha (0.05), dividing by the number of covariates (16), leaving 0.0031 as the new alpha to determine significance for the analyses. Post-hoc power analyses from the imputed multiple linear regression R^2^ are found within **Supplementary Table 4**.

Throughout the study periods, gender, age and type of diagnosis were not found to be significantly associated with ∆HAM-D. Improved ∆HAM-D 17 was found in patients taking tricyclic antidepressants (0–4 weeks: B = 4.85, p = 0.0002; 0–2 weeks: B = 3.58, p = 0.002) compared to patients taking SSRIs. Further significant associations were not found within the other antidepressants studied in the cohort.

### 5-HT Receptor Associations

No significant associations for 5-HTRs were identified in the cohort within all three time periods (**Table 3**, **Supplementary Table 3**). Of the SNPs identified to be significant in the univariate analyses, *HTR2C* rs5946189, *HTR3A* rs33940208 and *HTR3B* rs1176744 did not result in significance in the multiple linear regression.

**Table 3:** Multiple linear regression of total depression cohort covariates (age, gender, diagnosis, type of antidepressant, selected 5-HT receptor gene genotypes) between the four week and week two to four study period.

**∆HAM-D**	**Total study – 4 weeks**			**Weeks 2–4**		
**Dependent variables**	*B*	*95% CI of B*	*p*	*B*	*95% CI of B*	*p*
Gender						
Female	1.97	-0.65–4.59	0.14	2.02	-0.14–4.17	0.07
Age	-0.01	-0.1–0.07	0.74	-0.01	-0.08–0.05	0.68
Type of Diagnosis						
Recurrent	-1.62	-3.4–0.16	0.08	-0.29	-1.75–1.18	0.70
Antidepressant						
TCAs	4.85	2.3–7.4	0.0002**	1.27	-0.83–3.37	0.23
SNRIs	1.45	-1.75–4.65	0.37	1.06	-1.57–3.7	0.43
NaSSAs	-3.53	-7.07–0.01	0.05	-3.10	-6.01–-0.18	0.04
Agomelatine	1.24	-2.07–4.56	0.46	-2.06	-4.79–0.67	0.14
*HTR 1A*						
rs6295 C	3.56	-0.61–7.72	0.09	2.82	-0.6–6.25	0.11
rs6295 GC	3.78	-0.9–8.47	0.11	2.65	-1.21–6.5	0.18
rs749099 G	0.57	-3.58–4.71	0.79	1.51	-1.9–4.92	0.38
rs749099 CG	-3.14	-8.06–1.77	0.21	-1.46	-5.51–2.58	0.48
*HTR 1B*						
rs6296 C	-1.43	-4.62–1.76	0.38	-0.95	-3.58–1.67	0.47
rs6296 CG	-1.77	-4.97–1.44	0.28	-0.99	-3.63–1.65	0.46
*HTR 2A*						
rs7997012 G	-2.95	-5.64–0.26	0.03	-2.39	-4.6–-0.18	0.04
rs7997012 AG	-2.52	-4.67–0.37	0.02	-2.04	-3.81–-0.27	0.02
rs1928040 G	0.01	-3.15–3.17	0.99	-0.14	-2.74–2.46	0.92
rs1928040 GA	-0.58	-3.39–2.23	0.68	1.39	-0.92–3.7	0.24
rs6312 T	4.34	-0.46–9.15	0.08	4.81	0.85–8.76	0.02
rs6312 TC	2.96	-2.67–8.58	0.30	3.45	-1.18–8.07	0.14
*HTR 2C*						
rs5946189 TCC	4.35	-0.91–9.62	0.10	2.41	-1.93–6.74	0.27
rs6318 C	0.92	-4.96–6.8	0.76	-1.74	-6.58–3.1	0.48
rs6318 GC	-3.53	-8.5–1.43	0.16	-1.62	-5.7–2.47	0.43
rs17326429 AAG	-0.75	-2.88–1.38	0.49	-1.45	-3.2–0.3	0.10
*HTR 3A*						
rs33940208 TCC	-3.97	-8.41–0.48	0.08	-3.72	-7.37–--0.06	0.05
*HTR 3B*						
rs1176744 ACC	-0.19	-2.21–1.82	0.85	-1.45	-3.11–0.2	0.09
*HTR 6*						
rs1805054 TTC	1.12	-0.8–3.04	0.25	1.37	-0.21–2.95	0.09

Data is presented as regression coefficients (B), 95% confidence intervals (CI) and total explained variance (r^2^); Significance for *p* values after correction: p < 0.0031; ** p < 0.001; ΔHAM-D, Hamilton Depression Score Rating Difference; TCAs, tricyclic antidepressants; SNRIs, serotonin–norepinephrine reuptake inhibitors; NaSSAs, noradrenergic and specific serotonergic antidepressants.

Within the studied 5-HTR SNPs, suggestive associations were found within the total study and in the last 2 weeks, albeit not significant to the Bonferroni correction. Of note, *HTR1A* rs6295 homozygous C and heterozygous GC patients were found to indicate increased ∆HAM-D 17 compared to homozygous G patients. *HTR2A* rs6312 homozygous T and heterozygous TC patients illustrated a positive ∆HAM-D compared to homozygous C patients. *HTR2C* rs5946189 combined homozygous C and heterozygous TC patients suggest a positive ∆HAM-D compared to homozygous T patients. On the other hand, *HTR2A* rs7997012 homozygous C and heterozygous CG patients were indicative of decreased ∆HAM-D 17 when compared to homozygous G. *HTR3A* rs33940208 combined homozygous C and heterozygous TC patients point to a decreased ∆HAM-D 17 when compared to homozygous T. The remaining studied 5-HTR SNPs (*HTR1A* rs749099, *HTR1B* rs6296, *HTR2A* rs1928040, *HTR2C* rs6318, *HTR2C* rs17326429, *HTR3B* rs1176744, *HTR6* rs1805054) did not suggest a direction of ∆HAM-D inclination in the total study, nor in the last 2 weeks.

### Odds Ratio of Hemizygous 5-HT2C

Odds ratio (OR) was calculated for 5-HT2C genotypes to explore its hemizygous nature and to determine prevalence of depression. Depressed patients were compared against a control group of depression free individuals. In both total cohort and female-only OR comparisons, no significance was found for 5-HT2C receptor SNPs (**Table 4**). Male-only OR comparison was unable to be conducted due to the small sample size within the depression cohort.

**Table 4:** Odds ratio (OR) and 95% confidence interval (CI) for 5-HT2C receptors SNPs and depression.

a.	Total Cohort		
	**5-HT2C SNP (rs number)**	**OR**	**95% CI**
	rs3813929	1.12	0.4–3.13
	rs569959	0.71	0.35–1.45
	rs5946189	2.17	0.65–7.26
	rs4911871	2.32	0.95–5.68
	rs6318	1.32	0.49–3.59
	rs12858300	0.88	0.12–6.34
	rs17326429	0.85	0.3–2.36
	rs1801412	0.85	0.12–6.13
**b.**	**Female patients only**		
	**5-HT2C SNP (rs number)**	**OR**	**95% CI**
	rs3813929	1.09	0.19–6.16
	rs569959	0.61	0.2–1.83
	rs5946189	–	–
	rs4911871	4.84	1.23–19.1
	rs6318	3.24	0.37–28.32
	rs12858300	–	–
	rs17326429	0.86	0.15–4.87
	rs1801412	0.57	0.04–9.33

Odds ratio for 5-HT2C Receptor SNP between Depressed Cohort and Control Cohort; No significance found between Depressed Cohort and Control Cohort.

## Discussion

Due to the complex nature of depression, identifying a single etiology or pharmacological target for antidepressant treatment has been unconvincing. The monoamine theory stipulates neurotransmission dysfunction of the adrenergic/serotonergic pathways causes a depressive episode, providing a target for antidepressant treatment (Loonen and Ivanova, 2016). 5-HT receptor signaling may provide insight into its role in mediating antidepressant response, such as *via* the neural circuitry of the hippocampus (Yohn et al., 2017). However, no significant associations were found in our study within the selected 5-HT receptor polymorphisms on antidepressant response. This may stem from the different classes of antidepressants prescribed but as the majority of treatment prescribed are serotonin reuptake inhibitors, the lack of any associations in our cohort does not lend credence to the monoamine theory. On the other hand, the indications found within *HTR1A* rs6295, *HTR2A* rs6312, *HTR2A* rs7997012, *HTR2C* rs5946189 and *HTR3A* rs33940208 provide a potential direction for future studies to focus on targeting a specified 5-HT receptor and antidepressants whose mechanism of action target it.

Our results fall in line with previous literature and pharmacogenetic studies targeting 5-HT receptors, as results were inconclusive with indications toward antidepressant response and other mental health disorders (Serretti et al., 2009; Bondy, 2011; Reynolds et al., 2014). While there are some indicative associations within the selected 5-HT receptor polymorphisms, until further investigation with antidepressants targeting the specific 5-HT receptor are conducted, whether there are significant associations will not be known. We decided not to include any *HTR2B* SNPs in our investigation as its role as classical neurotransmitter receptor has not been clear. Until demonstrated recently, studies failed to demonstrate the presence of 5-HT2B receptors in the brain, whereas now several regions have been identified within the central nervous system (Devroye et al., 2018). 5-HT2B receptors appear to regulate the activity of serotonin transporters, by phosphorylation of the transporters, and may regulate the function of neuroglia, i.e. astrocytes, microglia, etc. (Devroye et al., 2018). This suggestive involvement of *HTR2B* in the neuroplastic process provides an interesting opportunity to study the antidepressant effects of SSRIs related to *HTR2B*-associated neuroplastic modulation, in combination with other neuroplastic factors (*BDNF*), to determine whether a potential relationship to antidepressant treatment response can be identified.

### Curious Nature of *HTR2C* and Depression in Males

The hemizygous nature of *HTR2C* in males presents an opportunity to investigate whether a genotype affects 5-HT2C receptor activity. Males are unable to be heterozygous for 5-HT2C receptor genotypes since the gene is X-linked, therefore, 5-HT2C genotypes affect males differently than in females. We looked to explore this within our male depressive cohort and determine whether an effect on antidepressant response could be found. However, the small sample size of male depressed patients in our study limited the possibility to investigate the potential pharmacogenetic effect of 5-HT2C receptor genotypes on antidepressant response.

The small number of male depressive patients in our study falls in line with the current prevalence of depression found in males, in comparison to females (Keuhner, 2017). Depression in males is often not investigated which may stem from the underreporting of depressive symptoms, due to a lack of understanding of their symptoms and perceived emasculation of reporting depression (Sigmon et al., 2005). As we look to develop an understanding on personalizing treatment for major depressive disorder, consideration on the effect of gender on the etiology of depression, and subsequent treatment, is required.

### Strengths and Limitations

Due to the recurrent nature of depression, exposure from previous treatments may hinder the effectiveness of following treatments. Our cohort consisted of treatment-free patients, thus the potential limiting effect of previous antidepressant treatment exposures (notably SSRI) is limited. Majority of our patients were prescribed serotonin reuptake inhibitors, providing the opportunity to study the effect of 5-HT receptor SNPs on treatment response.

The small number of patients studied in our cohort limited the number of covariates included in our final multiple regression analysis. In addition, the number of antidepressants prescribed to patients varied, limiting the possibility of stratifying the analysis per treatment. Therefore, investigating the mechanism of action of selected antidepressants targeting specific 5-HT receptors was not possible, limiting the scope of the study.

### Conclusion and Future Research

Over the course of study, significant associations between 5-HT receptors SNPs and antidepressant response were not identified. Our hypothesis of targeting the ‘true’ antidepressant effect within the last 2 weeks of the study similarly did not result in associations between the SNPs and treatment response. Currently, the first-line of antidepressant treatment for major depressive disorders are SSRIs but our investigation suggests tricyclic antidepressants are more effective as patients were found to respond with greater improvement in HAM-D 17 score over the course of the study.

Sub-analyses of treatment options were limited as the study size was not large enough. Therefore, future studies will need to consider narrowing treatments given, focusing on a specific mechanism of action and whether polymorphisms within the targeted 5-HT receptors will affect antidepressant response. Specifically, HTR2C provides a unique opportunity to explore tailored treatment for male patients due to the hemizygous nature of the gene.

## Author’s Note

This study was a collaboration between the Mental Health Research Institute (Tomsk National Research Medical Center of the Russian Academy of Sciences) in Tomsk and the Groningen Research Institute of Pharmacy (GRIP) of the University of Groningen. The Russian part is carried out within the framework of Tomsk Polytechnic University Competitiveness Enhancement Program.

## Data Availability Statement

The datasets generated for this study are available on every reasonable request to Prof. Dr. Svetlana A. Ivanova (ivanovaniipz@gmail.com), following approval of the Board of Directors of the MHRI, in line with local guidelines and regulations.

## Ethics Statement

The studies involving human participants were reviewed and approved by the Institutional Medical Review Board of the Mental Health Research Institute (protocol 49 from 23.04.12). The patients/participants provided their written informed consent to participate in this study.

## Author Contributions

TO and NV share first authorship. AL and SI instigated and designed the study. AL, BW and SI coordinated and supervised the study. TO, NV and IL designed and performed the statistical analysis and contributed to writing the paper. SI wrote the study protocol and selected the SNPs. IL monitored the study. GS collected clinical data. IL, NV, DP and IP isolated DNA, genotyped the samples and recorded all data in an Excel database. NB supervised the clinical work. SI, AL and BW supervised the technical work. TO and AL wrote the manuscript. GS, BW and SI adapted the manuscript. All authors read the paper and agree with its content. Their role justifies their (co)-authorship to this paper.

## Funding

Obtaining reagents for genotyping of 5-HT receptor genes was financed by the Russian Foundation for Basic Research (RFBR) grant №17-29-02205 «Development of a molecular genetic panel of depressive disorders based on polymorphisms of the genes of neuronal kinases, neurotrophic proteins and genes of the serotonergic system» and was also used for the salary payments of the Russian authors.

## Conflict of Interest

The authors declare that the research was conducted in the absence of any commercial or financial relationships that could be construed as a potential conflict of interest.
