# Supplementary material for: Limited Associations Between 5-HT Receptor Gene Polymorphisms and Treatment Response in Antidepressant Treatment-Free Patients With Depression
Source: Front Pharmacol. 2019 Dec 19;10:1462. doi: 10.3389/fphar.2019.01462 (PMC6951408; doi:10.3389/fphar.2019.01462)
Supplement: Supplementary file 1 [file DataSheet_1.docx]

**Supplementary Table 1 – 5-HT Receptor Subfamily and SNPs genotyped**

| **Gene** | **Chromosome** | **Locus** | **SNP** | **Minor Allele** | **MAF^1^** |
| --- | --- | --- | --- | --- | --- |
| HTR1A | 5 | q12.3 | rs6295 | G | 0.453 |
|  |  |  | rs1364043 | G | 0.331 |
|  |  |  | rs10042486 | T | 0.354 |
|  |  |  | rs749099 | C | 0.352 |
| HTR1B | 6 | q14.1 | rs6298 | A | 0.341 |
|  |  |  | rs6296 | G | 0.338 |
|  |  |  | rs130058* | A | 0.184 |
| HTR2A | 13 | q14.2 | rs6311 | T | 0.443 |
|  |  |  | rs6313 | A | 0.441 |
|  |  |  | rs6314 | A | 0.075 |
|  |  |  | rs7997012 | A | 0.273 |
|  |  |  | rs1928040 | G | 0.449 |
|  |  |  | rs9316233* | G | 0.298 |
|  |  |  | rs2224721* | T | 0.337 |
|  |  |  | rs6312 | C | 0.081 |
| HTR2C | X | q23 | rs3813929* | T | 0.135 |
|  |  |  | rs569959 | C | 0.313 |
|  |  |  | rs5946189* | C | 0.191 |
|  |  |  | rs4911871 | A | 0.172 |
|  |  |  | rs6318 | C | 0.166 |
|  |  |  | rs12858300* | C | 0.29 |
|  |  |  | rs17326429* | A | 0.135 |
|  |  |  | rs1801412* | C | 0.063 |
| HTR3A | 11 | q23.2 | rs1062613* | T | 0.248 |
|  |  |  | rs33940208* | T | 0.141 |
|  |  |  | rs1176713* | G | 0.277 |
| HTR3B | 11 | q23.2 | rs1176744* | C | 0.354 |
| HTR6 | 1 | p36.13 | rs1805054* | T | 0.173 |

MAF: minor allele frequency; MAF values taken from the 1000 Genomes Project; *Minor allele + heterozygous patients were combined in univariate analysis due to small number of patients who were homozygous for minor allele

**Supplementary Table 2 – Univariate analysis of 5-HT SNPs for HAM-D between week two and week four and four week study period**

|  |  | **Total study - four weeks** | | | **Week two to four** | | |
| --- | --- | --- | --- | --- | --- | --- | --- |
| **Variable**  **(5-HTR SNP)** | | *Beta* | *95% CI of Beta* | *P* | *Beta* | *95% CI of Beta* | *P* |
| 5-HTR 1A | |  |  |  |  |  |  |
|  | rs6295 | 0.87 | -0.38 - 2.13 | 0.17 | 0.52 | -0.51 - 1.55 | 0.32 |
|  | rs1364043 | -0.65 | -2.02 - 0.72 | 0.35 | -0.34 | -1.46 - 0.78 | 0.55 |
|  | rs10042486 | 0.66 | -0.56 - 1.89 | 0.29 | 0.39 | -0.62 - 1.41 | 0.44 |
|  | rs749099 | -0.73 | -1.97 - 0.49 | 0.24 | -0.31 | -1.33 - 0.71 | 0.55 |
| 5-HTR 1B | |  |  |  |  |  |  |
|  | rs6298 | 0.53 | -0.93 - 1.98 | 0.48 | -0.02 | -1.23 - 1.18 | 0.97 |
|  | rs6296 | -0.42 | -1.87 - 1.03 | 0.57 | 0.05 | -1.14 - 1.25 | 0.93 |
|  | rs130058 | 0.58 | -1.29 - 2.47 | 0.54 | 0.58 | -0.95 - 2.11 | 0.45 |
| 5-HTR 2A | |  |  |  |  |  |  |
|  | rs6311 | -0.23 | -1.56 - 1.09 | 0.73 | -0.55 | -1.65 - 0.54 | 0.32 |
|  | rs6313 | -0.16 | -1.49 - 1.16 | 0.81 | 0.10 | -0.99 - 1.21 | 0.85 |
|  | rs6314 | 0.1 | -2.78 - 2.99 | 0.94 | 0.81 | -1.45 - 3.08 | 0.48 |
|  | rs7997012 | -0.55 | -1.88 - 0.77 | 0.41 | -0.68 | -1.78 - 0.42 | 0.22 |
|  | rs1928040 | 0.42 | -0.93 - 1.78 | 0.54 | -0.49 | -1.61 - 0.63 | 0.39 |
|  | rs9316233 | -0.24 | -2.04 - 1.55 | 0.79 | 0.08 | -1.33 - 1.49 | 0.91 |
|  | rs2224721 | -0.51 | -2.09 - 1.07 | 0.53 | -0.56 | -1.87 - 0.73 | 0.39 |
|  | rs6312 | 0.78 | -1.91 - 3.49 | 0.57 | 1.53 | -0.69 - 3.76 | 0.18 |
| 5-HTR 2C | |  |  |  |  |  |  |
|  | rs3813929 | -0.41 | -1.89 - 1.06 | 0.58 | -0.69 | -1.91 - 0.52 | 0.26 |
|  | rs569959 | -0.54 | -1.70 - 0.61 | 0.36 | 0.19 | -0.77 - 1.15 | 0.70 |
|  | rs5946189 | 1.57 | 0.02 - 3.12 | 0.046* | 0.25 | -1.04 - 1.54 | 0.70 |
|  | rs4911871 | 0.27 | -1.21 - 1.77 | 0.71 | 0.37 | -0.86 - 1.61 | 0.55 |
|  | rs6318 | 1.28 | -0.23 - 2.80 | 0.10 | 0.07 | -1.20 - 1.35 | 0.91 |
|  | rs12858300 | 0.54 | -1.79 - 2.88 | 0.65 | 0.20 | -1.73 - 2.13 | 0.84 |
|  | rs17326429 | 0.44 | -1.07 - 1.95 | 0.57 | -0.77 | -0.48 - 2.02 | 0.23 |
|  | rs1801412 | 0.17 | -2.08 - 2.43 | 0.88 | 0.28 | -1.58 - 2.15 | 0.77 |
| 5-HTR 3A | |  |  |  |  |  |  |
|  | rs1062613 | 0.5 | -1.08 - 2.10 | 0.53 | 0.31 | -0.93 - 1.57 | 0.62 |
|  | rs33940208 | 4.39 | -0.06 - 8.84 | 0.05 | 4.34 | 0.68 - 8.00 | 0.020* |
|  | rs1176713 | 0.66 | -0.96 - 2.29 | 0.42 | 0.37 | -0.97 - 1.72 | 0.58 |
| 5-HTR 3B | |  |  |  |  |  |  |
|  | rs1176744 | -0.35 | -2.38 - 1.67 | 0.73 | -1.71 | -3.36 - -0.06 | 0.041* |
| 5-HTR 6 | |  |  |  |  |  |  |
|  | rs1805054 | -0.92 | -2.50 - 0.65 | 0.25 | -1.12 | -2.43 - 0.17 | 0.09 |

Table denoting univariate analysis of 5-HT Receptor SNPs for selection process for multiple linear regression; **p* < 0.05

**Supplementary Table 3 – Multiple linear regression of total depression cohort covariates (age, gender, diagnosis, type of antidepressant, selected 5-HTR genotypes) between initiation and week two**

| **∆HAM-D** | | **Initiation to week two** | | |
| --- | --- | --- | --- | --- |
| **Dependent variables** | | *Beta* | *95% CI of Beta* | *p* |
| Gender | |  |  |  |
|  | Female | -0.05 | -2.38 – 2.29 | 0.97 |
| Age | | 0.00 | -0.07 – 0.07 | 1.00 |
| Type of Diagnosis | |  |  |  |
|  | Recurrent | -1.33 | -2.92 – 0.26 | 0.10 |
| Antidepressant | |  |  |  |
|  | TCAs | 3.58 | 1.31 – 5.85 | 0.002* |
|  | SNRIs | 0.39 | -2.46 – 3.24 | 0.79 |
|  | NaSSAs | -0.43 | -3.59 – 2.72 | 0.79 |
|  | Agomelatine | 3.31 | 0.35 – 6.26 | 0.03 |
| HTR 1A | |  |  |  |
|  | rs6295 C | 0.74 | -2.98 – 4.44 | 0.70 |
|  | rs6295 GC | 1.14 | -3.04 – 5.31 | 0.59 |
|  | rs749099 G | -0.94 | -4.63 – 2.75 | 0.62 |
|  | rs749099 CG | -1.68 | -6.06 – 2.7 | 0.45 |
| HTR 1B | |  |  |  |
|  | rs6296 C | -0.48 | -3.31 – 2.36 | 0.74 |
|  | rs6296 CG | -0.78 | -3.63 – 2.08 | 0.59 |
| HTR 2A | |  |  |  |
|  | rs7997012 G | -0.56 | -2.95 – 1.84 | 0.65 |
|  | rs7997012 AG | -0.48 | -2.4 – 1.44 | 0.62 |
|  | rs1928040 G | 0.15 | -2.66 – 2.96 | 0.92 |
|  | rs1928040 GA | -1.97 | -4.48 – 0.53 | 0.12 |
|  | rs6312 T | -0.46 | -4.74 – 3.82 | 0.83 |
|  | rs6312 TC | -0.49 | -5.5 – 4.52 | 0.85 |
| HTR 2C | |  |  |  |
|  | rs5946189 TCC | 1.95 | -2.74 – 6.64 | 0.41 |
|  | rs6318 C | 2.66 | -2.58 – 7.9 | 0.32 |
|  | rs6318 GC | -1.91 | -6.34 – 2.51 | 0.39 |
|  | rs17326429 AAG | 0.70 | -1.2 – 2.6 | 0.47 |
| HTR 3A | |  |  |  |
|  | rs33940208 TCC | -0.25 | -4.21 – 3.71 | 0.90 |
| HTR 3B | |  |  |  |
|  | rs1176744 ACC | 1.26 | -0.54 – 3.05 | 0.17 |
| HTR 6 | |  |  |  |
|  | rs1805054 TTC | -0.25 | -1.96 – 1.46 | 0.77 |

Data is presented as regression coefficients (B), 95% confidence intervals (CI) and total explained variance (r^2^); Significance for *p* values after correction: * *p* < 0.0031; HAM-D: Hamilton Depression Score Rating Difference; TCAs: tricyclic antidepressants; SNRIs: serotonin–norepinephrine reuptake inhibitors; NaSSAs: noradrenergic and specific serotonergic antidepressants

**Supplementary Table 4 – Power analysis for multiple linear regression for the three time periods**

|  |  |  | Effect size (R^2^) | Power  (1- β error probability) | Critical F |
| --- | --- | --- | --- | --- | --- |
| Total study - four weeks | | | 0.278 | 0.925 | 2.416 |
| Week two to four | | | 0.283 | 0.931 | 2.416 |
| Initiation to week two | | | 0.202 | 0.735 | 2.416 |

Power analysis calculations from G*Power; F-Test, linear multiple regression, deviation from zero, Post-hoc; Sample size: 156; Variable number; 16
